# Alchemical Free-Energy Calculations of Watson–Crick and Hoogsteen Base Pairing Interconversion in DNA

**DOI:** 10.1021/acs.jctc.2c00848

**Published:** 2022-10-06

**Authors:** Inacrist Geronimo, Marco De Vivo

**Affiliations:** Laboratory of Molecular Modelling & Drug Discovery, Istituto Italiano di Tecnologia, Via Morego 30, Genoa 16163, Italy

## Abstract

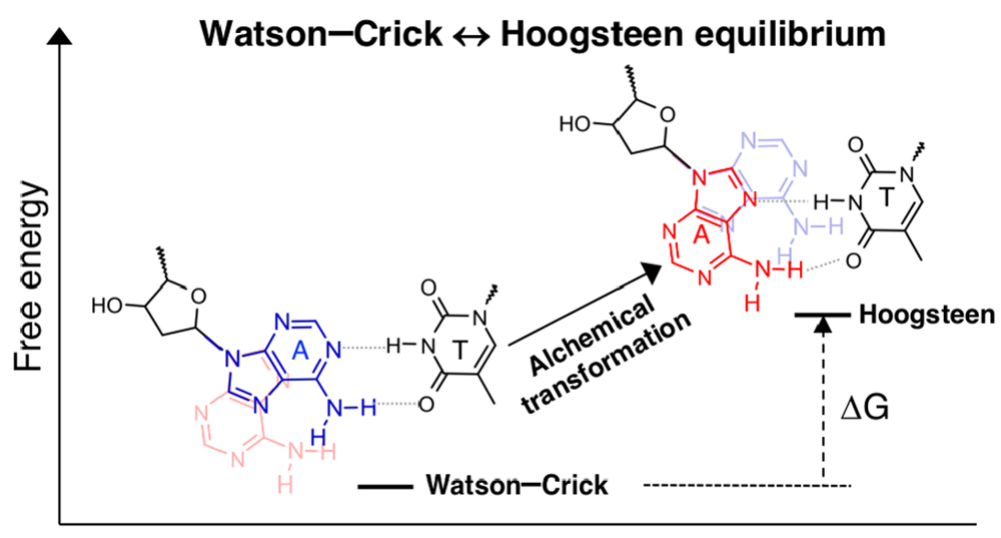

Hoogsteen (HG) base pairs have a transient nature and can be structurally similar to Watson–Crick (WC) base pairs, making their occurrence and thermodynamic stability difficult to determine experimentally. Herein, we employed the restrain–free-energy perturbation–release (R-FEP-R) method to calculate the relative free energy of the WC and HG base pairing modes in isolated and bound DNA systems and predict the glycosyl torsion conformational preference of purine bases. Notably, this method does not require prior knowledge of the transition pathway between the two end states. Remarkably, relatively fast convergence was reached, with results in excellent agreement with experimental data for all the examined DNA systems. The R-REP-R method successfully determined the stability of HG base pairing and more generally, the conformational preference of purine bases, in these systems. Therefore, this computational approach can help to understand the dynamic equilibrium between the WC and HG base pairing modes in DNA.

## Introduction

**Scheme 1:**
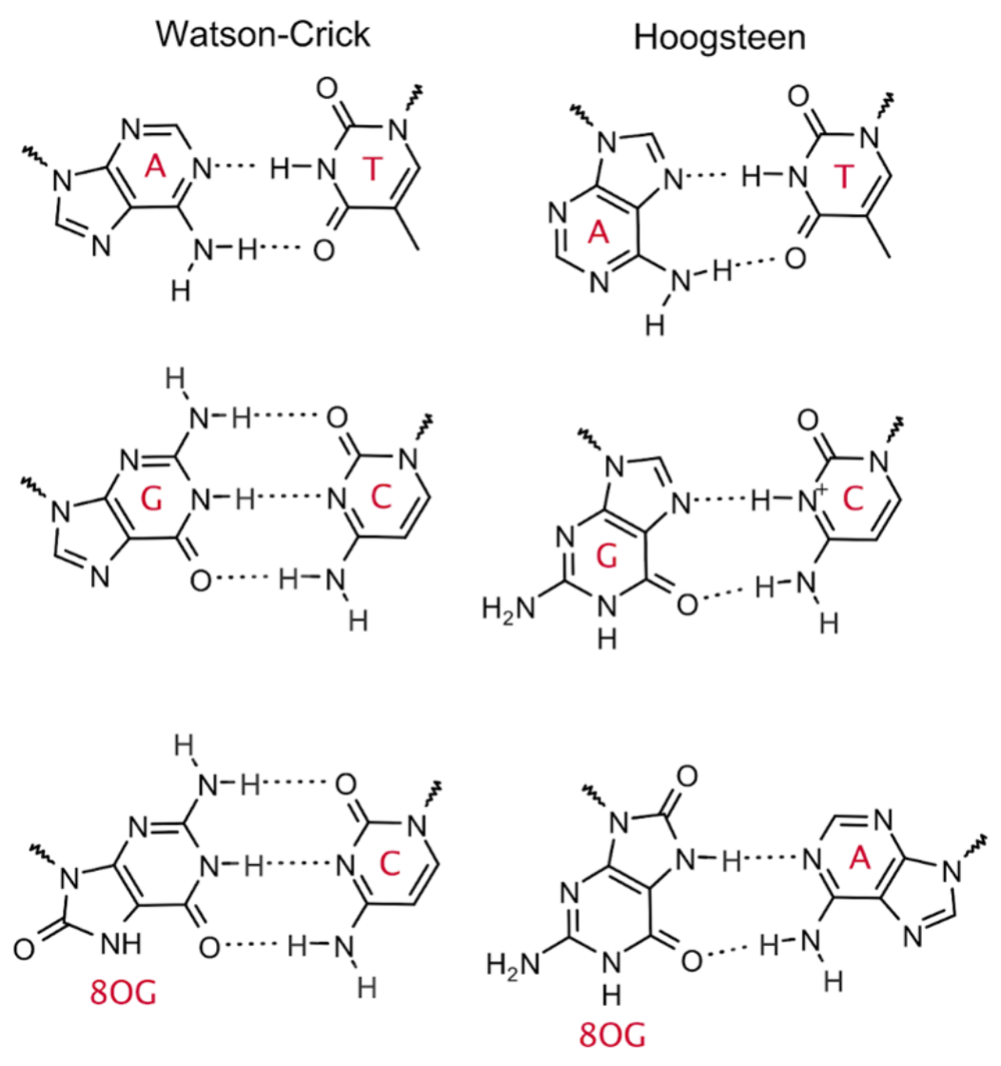
Watson–Crick and Hoogsteen Base Pairs

A defining feature of DNA is the canonical Watson–Crick (WC) base pairing of adenine (A)–thymine (T) and guanine (G)–cytosine (C). However, these bases can adopt an alternative base pairing mode known as Hoogsteen (HG) base pairing, in which the five-membered ring, instead of the six-membered ring, of the purine base (A or G) is hydrogen-bonded to the pyrimidine base (T or C) (Scheme 1). The transition between the two base pairing modes occurs via an *anti* → *syn* conformational change of the glycosyl torsion angle of the purine base [χ (O4′-C1′–N9-C4)]. Although less common than WC base pairing, HG base pairing is hypothesized to play important roles in replication by DNA polymerase (Pol) ι,^1,2^ recognition by transcription factors^3−6^ and DNA repair enzymes,^7−10^ and binding to small molecules.^11^

The work of Al-Hashimi et al. indicated a dynamic equilibrium between WC and HG base pairs in free DNA in solution, and, despite its transient nature (lifetimes of ∼1.5 and ∼0.3 ms for G:C^+^ and A:T, respectively), HG base pairing is thermodynamically stable, being only ∼3 kcal/mol higher in energy than the WC base pairing.^12^ The important implications of this thermal fluctuation (referred to as “DNA breathing”) on DNA recognition, binding, and damage repair^13−15^ has spurred mechanistic studies of the WC↔HG transition in DNA using different computational methods, including umbrella sampling,^16^ transition path sampling,^17^ metadynamics,^18−20^ and Markov state modeling.^18^ These computational studies provided an atomic-level description of the process, revealing a complex mechanism involving hydrogen bond breaking/formation between the base pairs, multiple pathways for purine base flipping toward the major or minor groove, and clockwise or counterclockwise rotation of the purine about the glycosidic bond.

While the aforementioned computational methods are a natural choice to gain mechanistic insights into the WC↔HG transition, using such methods to calculate the relative energy of the two base pairing modes in a wide range of systems (e.g., protein- or ligand-bound DNA and damaged DNA) can be quite challenging. This is because the complexity of the WC↔HG transition mechanism makes it difficult to identify the minimum energy pathway and determine the appropriate set of reaction coordinates (i.e., collective variables or CVs) that captures the slowest motions of the system.^18,21^ In the case of free DNA, although there appears to be a consensus that the most favorable WC↔HG transition pathway is via purine base flipping toward the major groove,^12,16−18^ different CVs have been used to calculate the relative free energy of the WC and HG base pairs. For example, Pak et al. employed a pseudodihedral angle describing base flipping, in addition to the glycosyl torsion angle of the purine, as a CV for 2D umbrella sampling.^16^ Swenson et al. used the function arctan2(*d*_WC_, *d*_HG_), where *d*_WC_ and *d*_HG_ are the distances of the N3 atom of thymine from the N1 and N7 atoms of adenine, respectively, for transition interface sampling (TIS).^17^ On the other hand, Ray and Andricioaei used the two slowest degrees of freedom obtained from time-lagged independent component analysis, which are strongly correlated with the hydrogen bond distances, phosphodiester bond torsion angles, and purine glycosyl torsion angle, to map the free-energy landscape.^18^

Thus, when one is only interested in the thermodynamic aspect of the WC↔HG transition (i.e., the free-energy difference), an alchemical approach, which does not require knowledge of the transition pathway and reaction coordinate(s) connecting the two end states, would be more advantageous than CV-based approaches, such as umbrella sampling and metadynamics. One such alchemical approach is the restrain–free-energy perturbation–release (R-FEP-R) method that was developed by Levy et al.^22^ to calculate conformational free-energy differences. The R-FEP-R method is based on the dual-topology FEP method^23−25^ for calculating the relative binding free energy of two ligands: atoms involved in the conformational change are removed from the initial conformational state and simultaneously grown back in the final conformational state in a series of steps controlled by the coupling parameter λ. Restraints are imposed on these atoms during the FEP calculation to maintain the initial or final conformational state and accelerate convergence, and the free-energy change due to the addition of these restraints is also calculated. The R-FEP-R method performed well against benchmarks of commonly used model systems, including alanine dipeptide, T4 lysozyme, and β-turn flip in ubiquitin.^22,26^

In this study, we used the R-FEP-R method coupled with the parmbsc1 force field^27^ to calculate the relative free energy of the WC and HG base pairs. We also assess the general applicability of this method in determining the conformational preference of purine bases in different systems. Specifically, we calculated the relative free energy of the WC and HG base pairing modes of an A:T base pair in a well-studied AT-rich DNA model system and relative free energy of the *anti* and *syn* glycosyl torsional conformations of an unpaired oxidized guanine (8-oxoguanine or 8OG) bound to a DNA repair enzyme, Pol μ. The results agreed well with experimental data, demonstrating that R-FEP-R/parmbsc1 is a simple yet accurate method of predicting the base-pairing and conformational preferences of purine bases in various contexts, including free, bound, mismatched, and damaged DNA.

## COMPUTATIONAL METHOD

### System Preparation and Equilibration

Relative free-energy calculations by the R-FEP-R method were performed for two systems: (1) an isolated AT-rich DNA and (2) binary 8OG-damaged DNA/Pol μ complex. For system 1, the nucleotide sequence was 5′-CGATTTTTTGGC-3′ (complementary strand 5′-GCCAAAAAATCG-3′). The adenine in the 4th position of the complementary strand (A4) was selected for the *anti*→*syn* conformational change leading to the WC↔HG conversion of the base pair with the thymine at the 9th position of the sequence (T9). An ideal B-DNA duplex structure for this sequence was built using the nucleic acid builder.^28^ Two models of system 1 were then prepared: one in which A4 is in the *anti* conformation and forms a WC base pair with T9, and the other in which A4 is in the *syn* conformation and forms a HG base pair with T9.

For system 2, the initial coordinates were taken from the crystal structure with Protein Databank (PDB) ID 6P1M.^29^ In this structure, the catalytic domain (P132–A434) has been truncated by replacing the disordered loop connecting β-strands 4 and 5 (loop 2, P398–P410) with Gly410 to improve crystallization.^29,30^ This modification was retained in our models since it does not significantly affect the gap-filling activity of Pol μ.^30^ On the other hand, missing residues in loop 1 (C369–F385) and the N-terminal end of the catalytic domain were built using Modeller 10.1.^31^ Two models of system 2 were then prepared: one in which 8-oxoguanine (8OG) is in *anti* conformation and the other in the *syn* conformation. In both cases, 8OG is unpaired, because the system is a binary complex without an incoming nucleotide bound in the Pol active site.

All systems were solvated in a rhombic dodecahedral box of TIP3P^32^ water, with a buffer distance of 12 Å between each wall and the closest atom in each direction. System 1 was neutralized by adding Na^+^ ions, and additional Na^+^ and Cl^–^ ions were added to achieve an ionic concentration of 25 mM NaCl, as in the experimental study of Al-Hashimi et al.^12^ For system 2, K^+^ ions were used instead of Na^+^ for neutralization, and additional Mg^2+^, K^+^, and Cl^–^ ions were added to achieve ionic concentrations of 50 mM KCl and 2.5 mM MgCl_2_. The protein and DNA were described using the AMBER14ffSB^33^ and parmbsc1^27^ force fields, respectively, which we have previously used to study the mechanisms of nucleic-acid-processing enzymes.^34,35^ The charges for 8OG (Table S1 in the Supporting Information) were derived by multiconformational restrained electrostatic potential fitting,^36,37^ as explained in more detail in the Supporting Information.

Minimization, heating, and equilibration of all systems are also described in the Supporting Information. One microsecond of unbiased MD simulation in the NPT ensemble was performed for each system (total of 4 μs) using GROMACS 2020.6.^38^

### R-FEP-R Calculations

#### Dual Topology and Restraints

**Figure 1:**
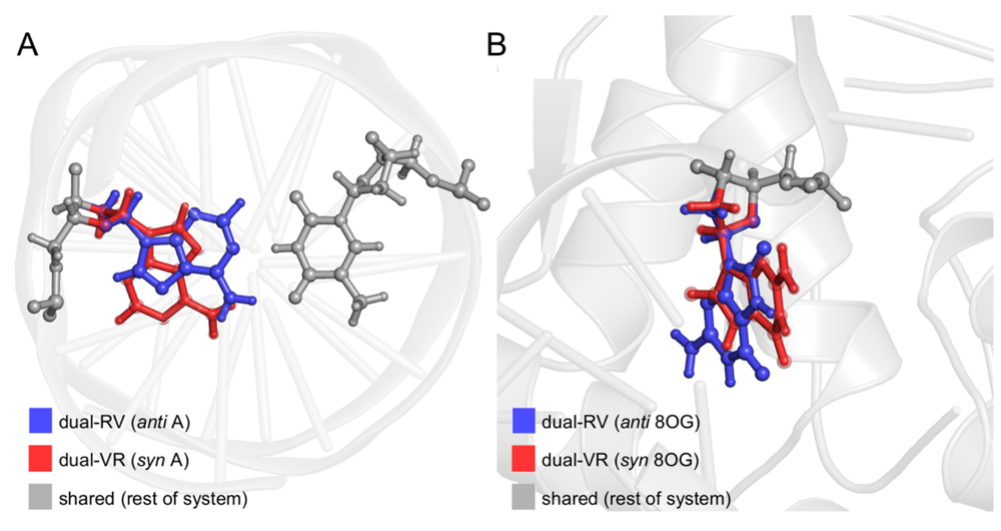
Hybrid models of (A) isolated AT-rich DNA and (B) binary 8-oxoguanine (8OG)-damaged DNA/polymerase μ complex. During the free-energy perturbation simulations, the dual-RV set changes from real to virtual, the dual-VR set changes from virtual to real, and the shared set does not change.

In the R-FEP-R method,^22^ the system is divided into three sets: (1) dual-RV set, which represents one conformation of the residue/fragment of interest that changes from real to virtual (i.e., dummy) during the FEP simulations, (2) dual-VR set, which represents the other conformation that changes from virtual to real, and (3) shared set, which is the rest of the system that does not change. In this study, all nonbackbone atoms of the purine base (A in system 1 and 8OG in system 2) were selected as the dual-RV(VR) set since they differ in position in the two conformations upon structural alignment (Figure 1). Hybrid models of the two systems, in which the purine base is simultaneously present in the *anti* (dual-RV set) and *syn* (dual-VR set) conformations, were built using the equilibrated structures from the unbiased MD simulations. Unlike the original study of Levy et al.,^22^ the proper dihedral potentials of the dual-RV(VR) set were always switched on because switching them off was observed to cause distortion of the purine ring, which is included in the set. Thus, only the van der Waals and Coulomb interactions were switched off (dual-RV set) or on (dual-VR set) during the FEP simulations. An excerpt of the GROMACS topology file is shown in Figure S1 in the Supporting Information to illustrate how the transformation from one conformation to the other is implemented in practice.

**Scheme 2:**
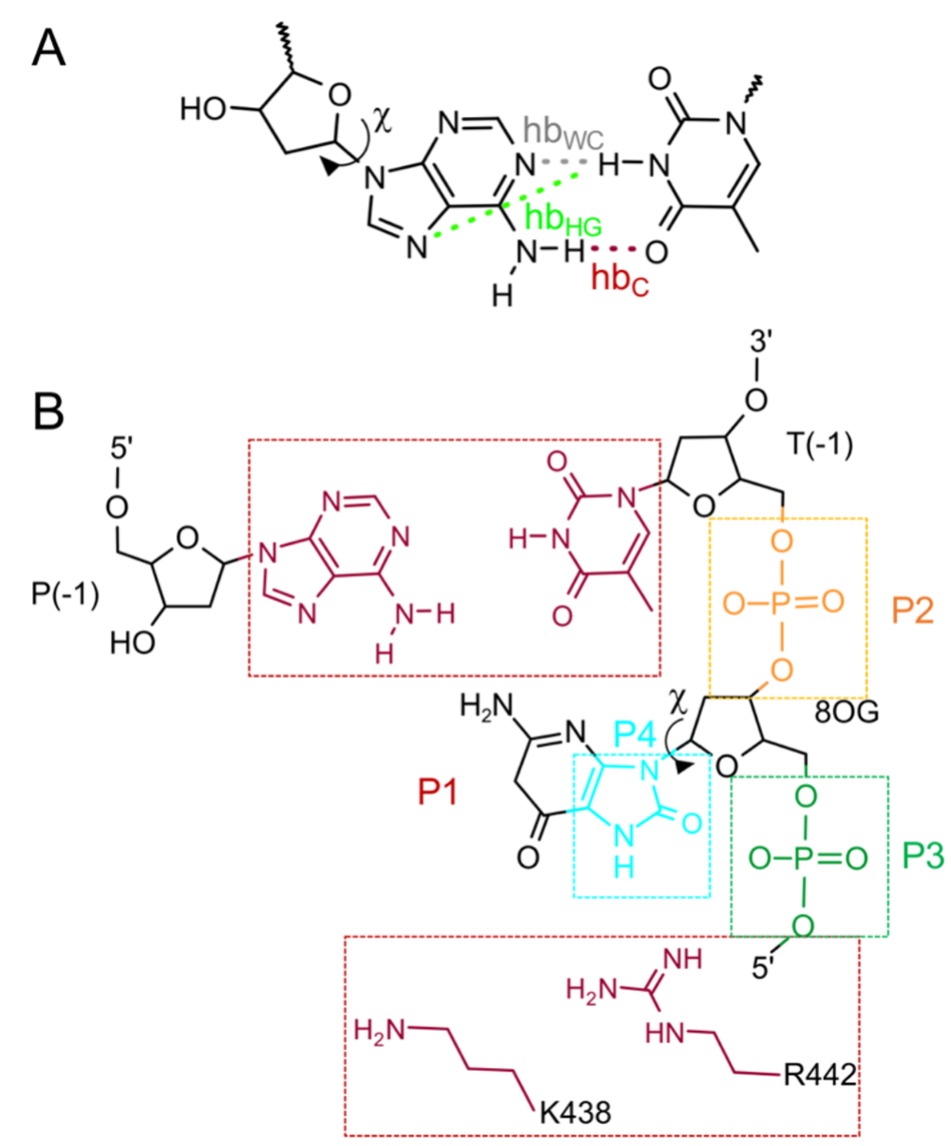
Harmonic Restraints for (A) Isolated AT-Rich DNA and (B) Binary 8-Oxoguanine (8OG)-Damaged DNA/Polymerase μ Complex χ, glycosyl torsion angle O4′-C1′–N9-C4; hb_WC_, hydrogen bond unique to Watson–Crick (WC) base pair; hb_HG_, hydrogen bond unique to Hoogsteen (HG) base pair; hb_C_, hydrogen-bond common to WC and HG base pairs. χ, glycosyl torsion angle O4′-C1′–N9-C4; base-flipping torsion angle P1–P2–P3-P4 based on the scheme originally proposed by Pak et al.;^16^ P1 (red), center of mass of the K438 and R442 side chains and T(−1) and P(−1) bases (excluding hydrogen); P2 (orange) and P3 (green), centers of mass of the T(−1) and 8OG phosphate groups, respectively; P4 (cyan), center of mass of the five-membered ring of 8OG (excluding hydrogen).

During the transformation, harmonic restraints were imposed on the glycosyl torsion angle using the [dihedral_restraints] directive in the GROMACS topology file to keep the purine base in either the *anti* or *syn* conformation (Scheme 2). Additional harmonic restraints were also used to prevent the purine base from flipping out of the helix and, in the case of system 1, to maintain the A4:T9 base-pair interactions, when the Coulomb and van der Waals interactions are not fully turned on. For system 1, harmonic restraints were placed on all hydrogen-bond distances and angles in the A4:T9 base pair (Scheme 2A) using the [intermolecular_interactions] directive in the GROMACS topology file. For system 2, since 8OG is unpaired, restraints were placed on a pseudodihedral angle (i.e., base-flipping angle, Scheme 2B) using the pull code in the GROMACS input file. The force constant for the harmonic restraints was 1000 kJ mol^–1^ rad^–2^ (dihedrals and angles) or 1000 kJ mol^–1^ nm^–2^ (distance), and the equilibrium values (obtained from the unbiased MD simulations) are summarized in Tables S2 and S3 in the Supporting Information.

#### Simulations and Postprocessing

**Scheme 3:**
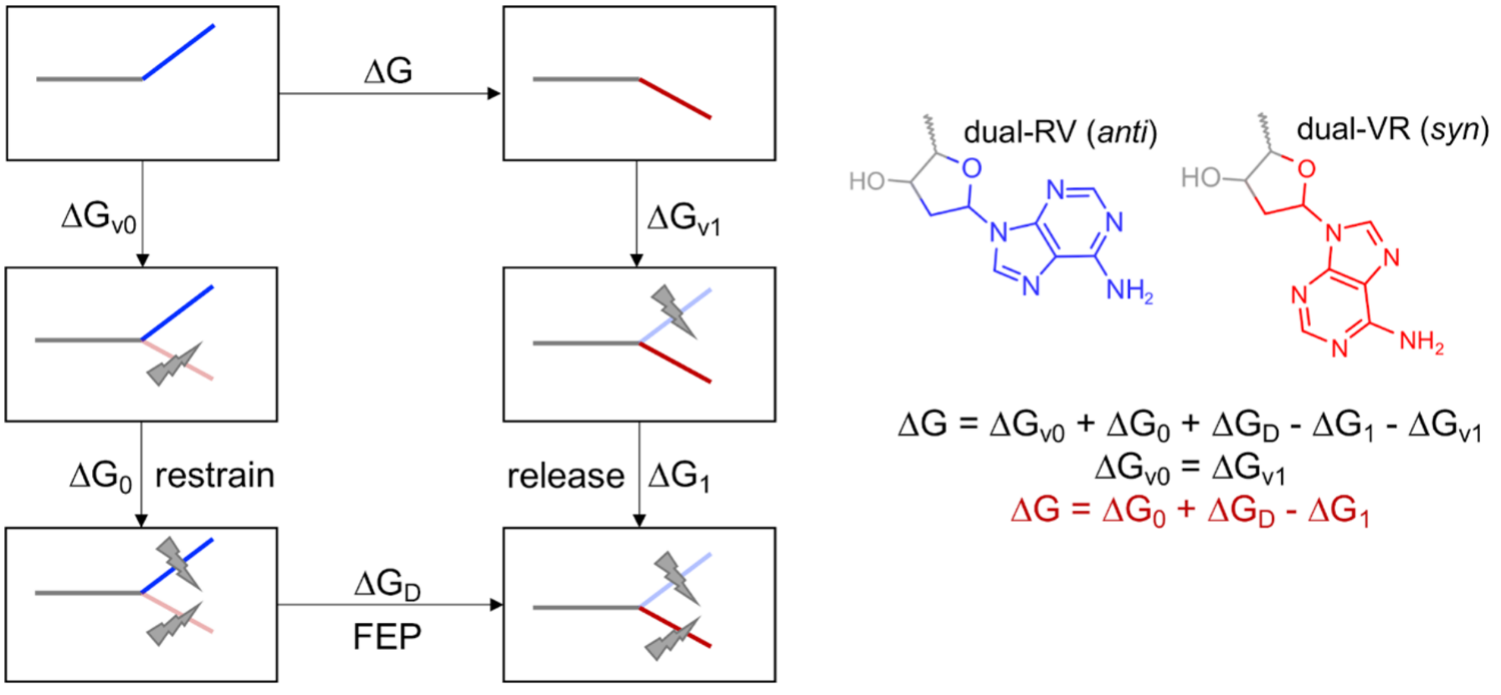
Thermodynamic Cycle for Calculating the Conformational Free-Energy Difference by the Restrain–Free Energy Perturbation–Release (R-FEP-R) Method

The thermodynamic cycle for calculating the conformational free-energy difference by the R-FEP-R method is illustrated in Scheme 3. The *anti* and *syn* conformations of the purine base (A or 8OG) were designated as the initial and final states, respectively. At the initial state, *anti* A/8OG is unrestrained with the Coulomb and van der Waals interactions switched on, while *syn* A/8OG is restrained with the Coulomb and van der Waals interactions switched off. *Anti* A/8OG is transformed to *syn* A/8OG in three stages:

(1) Restrain: The harmonic restraints on *anti* A/8OG are switched on using λ values of 0.0, 0.05, 0.25, 0.5, 0.75, and 1.0. The dihedral and pull code restraints are controlled by restraint-lambdas, and the intermolecular interactions by bonded-lambdas in the GROMACS input file.
(2) FEP: The Coulomb and van der Waals interactions of *anti* A/8OG are switched off, while those of *syn* A/8OG are switched on using λ values of 0.0, 0.01, 0.025, 0.05, 0.1, 0.2, 0.35, 0.5, 0.65, 0.8, 0.9, 0.95, 0.975, 0.99, and 1.0. The Coulomb and van der Waals interactions are controlled by coul-lambdas and vdw-lambdas, respectively, in the GROMACS input file.
(3) Release: The harmonic restraints on *syn* A/8OG are switched off using λ values of 1.0, 0.75, 0.5, 0.25, 0.05, 0.0. Thus, at the final state, *anti* A/8OG is restrained with the Coulomb and van der Waals interactions switched off, while *syn* A/8OG is unrestrained with the Coulomb and van der Waals interactions switched on.

The R-FEP-R calculations were performed using GROMACS 2020.5.^38^ Each λ-state was minimized for 1000 steps using the steepest descent algorithm, followed by the conjugate gradient algorithm, until the maximum force was less than 100 kJ mol^–1^ nm^–1^. Subsequently, each λ-state was heated to 300 K (system 1) or 310 K (system 2) for 100 ps in the NVT ensemble and equilibrated for 1 ns in the NPT ensemble with all heavy atoms restrained. Production simulation was run until the free energy converged. Constant temperature was maintained using Langevin dynamics^39^ with a time coupling constant of 1 ps. A constant pressure of 1 bar was maintained using the Berendsen algorithm^40^ during equilibration and the Parrinello–Rahman algorithm^41^ during production with a time coupling constant of 2 ps. Periodic boundary conditions were applied, and long-range electrostatic interactions were calculated using the particle mesh Ewald method^42^ with a real-space cutoff of 12 Å. Because the transformation included hydrogen atoms, bonds were not constrained, thereby necessitating a small time step of 0.5 fs. Soft-core potentials^43^ with a soft-core parameter (sc-alpha) of 0.5, soft-core power (sc-power) of 1, and soft-core radius (sc-sigma) of 0.3 nm were used for both Coulomb and van der Waals interactions to avoid singularity. The derivatives of the Hamiltonian with respect to λ (δ*H*/δλ) were written out every picosecond (2000 steps). The free energy for each stage of the transformation was calculated using the Multistate Bennett Acceptance Ratio method^44^ implemented in the alchemlyb Python library.^45^ The free-energy difference between WC and HG A4:T9 or *anti* and *syn* 8OG is the sum of the free energies for the restrain, FEP, and release stages (Scheme 3). Statistical, phase-space overlap, and convergence analyses were also performed using the alchemlyb Python library.

## Results and Discussion

### Isolated AT-Rich DNA

**Figure 2:**
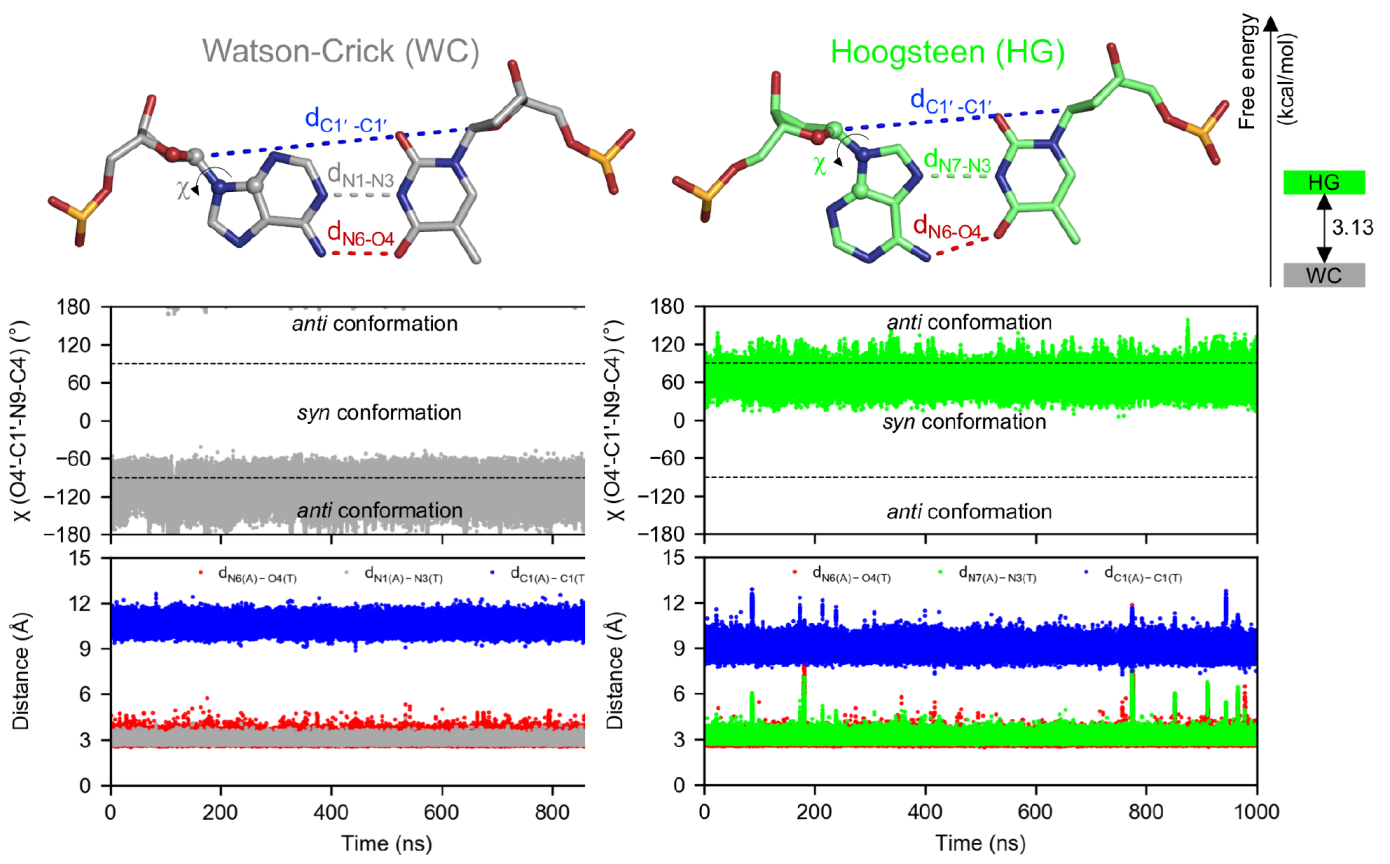
Watson–Crick and Hoogsteen base pairing modes of A4:T9 in isolated AT-rich DNA. The time evolution of the glycosyl torsion angle [χ (O4′-C1′–N9-C4), shown in ball-and-stick representation], C1′–C1′ distance (*d*_C1′_–_C1′_), and hydrogen-bond distances (*d*_N1–N3_, *d*_N6–O4_, and *d*_N7–N3_) during the 1-μs unbiased MD simulations is shown. The free-energy difference between the two base pairing modes calculated by the restrain–free-energy perturbation–release method is 3.13 ± 0.4 kcal/mol.

The WC and HG models are both structurally stable during the simulations (see Figure S2A in the Supporting Information), with the internal base pairs having backbone RMSDs of 1.7 and 1.6 Å, respectively. Overall, the two models are structurally similar with a backbone RMSD of 0.5 Å (Figure S2B in the Supporting Information). The only major structural difference is the shorter C1′–C1′ distance of the A4:T9 base pair in the HG model (*d*_C1′–C1′_ = 9.0 ± 0.3 Å compared with *d*_C1′–C1′_ = 10.6 ± 0.3 Å in the WC model; see Figure 2, as well as Figure S2B). Importantly, A4 maintains its *anti* conformation (χ = −105° ± 17°) and WC hydrogen bonds with T9 (*d*_N1–N3_ = 3.0 ± 0.1 Å and *d*_N6–O4_ = 3.0 ± 0.2 Å) throughout the simulation of the WC model; similarly, the *syn* conformation (χ = 64° ± 12°) and HG hydrogen bonds (*d*_N7–N3_ = 3.1 ± 0.2 Å and *d*_N6–O4_ = 2.9 ± 0.3 Å) are stable throughout the simulation of the HG model (Figure 2). In other words, no interconversion between the two base pairing modes occurs during the 1-μs-long simulations. These results also show that parmbsc1 is a suitable force field, at least for systems with only one HG base pair, despite earlier reports of structural distortion during the simulation of purely HG DNA systems.^27,46^

The equilibrated structures from these unbiased MD simulations were used to build a hybrid model with A4 simultaneously in the *anti* and *syn* conformations, which results in having both the WC and HG base pairing modes in the model (Figure 1A). For the R-FEP-R calculations, 6, 15, and 6 λ-states were used for the restrain, FEP, and release stages, respectively. Figure S3 shows that the chosen intervals are sufficient for phase-space overlap, with probabilities well above the recommended threshold of 0.03.^47^ Additionally, Figures S4–S9 in the Supporting Information show that the restraints on the glycosyl torsion angle and hydrogen-bond distances and angles (Scheme 2A) keep the dual-RV (*anti* A4) and dual-VR sets (*syn* A4) in their respective conformations and prevent their extrahelical movement.

**Figure 3:**
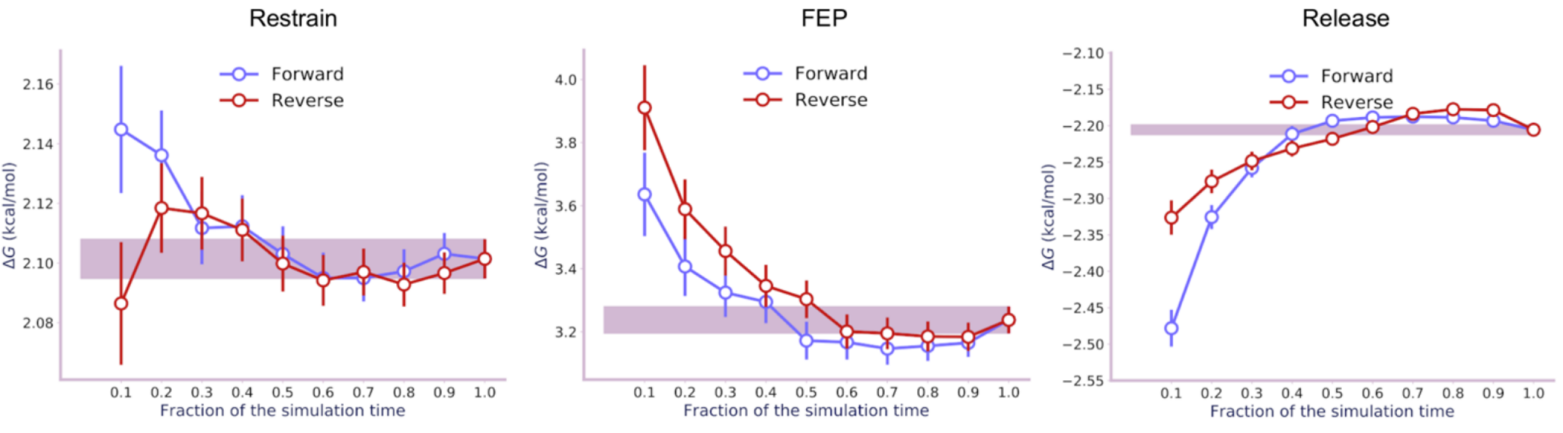
Time evolution of the free energies (with error bars) for the three stages of transformation of A4:T9 from Watson–Crick to Hoogsteen base pairing. Data from the last 10 ns of simulations were analyzed both chronologically (“forward”) and in a time-reversed manner (“reverse”).

The restrain, FEP, and release stages took 15, 21, and 18 ns to converge, respectively, which, multiplied by the number of λ-states per stage, led to a total simulation time of ∼0.5 μs. Convergence was confirmed by analyzing the simulation data in the forward and reverse directions and checking that the calculated free energies agree within error (Figure 3).^47^ For the same system, 2D umbrella sampling took 6 μs,^16^ while metadynamics/extended-system adaptive biasing force (meta-eABF) took 0.2 μs,^18^ for the free energy to converge.

Using the R-FEP-R method and parmbsc1 force field, the calculated free-energy difference between the WC and HG base pairing modes of A4:T9 is 3.13 ± 0.4 kcal/mol (Figure 2), which is in good agreement with the experimental value of 3.0–3.5 kcal/mol,^12^ obtained by thermodynamic analysis of NMR relaxation dispersion spectroscopy data. In comparison, calculated values of 3.2, 4.4, and 4.5 kcal/mol were obtained by TIS (Amber03 force field),^17^ 2D umbrella sampling (modified parmbsc0 force field),^16^ and meta-eABF (CHARMM36 force field),^18^ respectively. Thus, in terms of accuracy and computational cost, R-FEP-R coupled with parmbsc1 is an efficient method of calculating the relative free energy of the WC and HG base pairing modes. An additional advantage is that, unlike the CV-based methods mentioned above, R-FEP-R does not require knowledge of the transition pathway between the two base pairing modes. Thus, it can be easily applied in predicting the preferred base pairing mode for a wide range of free and bound DNA systems.

### Binary 8OG-Damaged DNA/Pol μ Complex

8OG, which is a common oxidation product of guanine, can adopt either the *anti* or *syn* conformation. The latter conformation enables the binding of the wrong base, adenine, via HG base pairing (Scheme 1), leading to misincorporation. One of the most error-prone Pols is Pol μ, which has an error frequency of ∼50 for the incorporation of cytosine vs adenine opposite template 8OG.^29^

**Figure 4:**
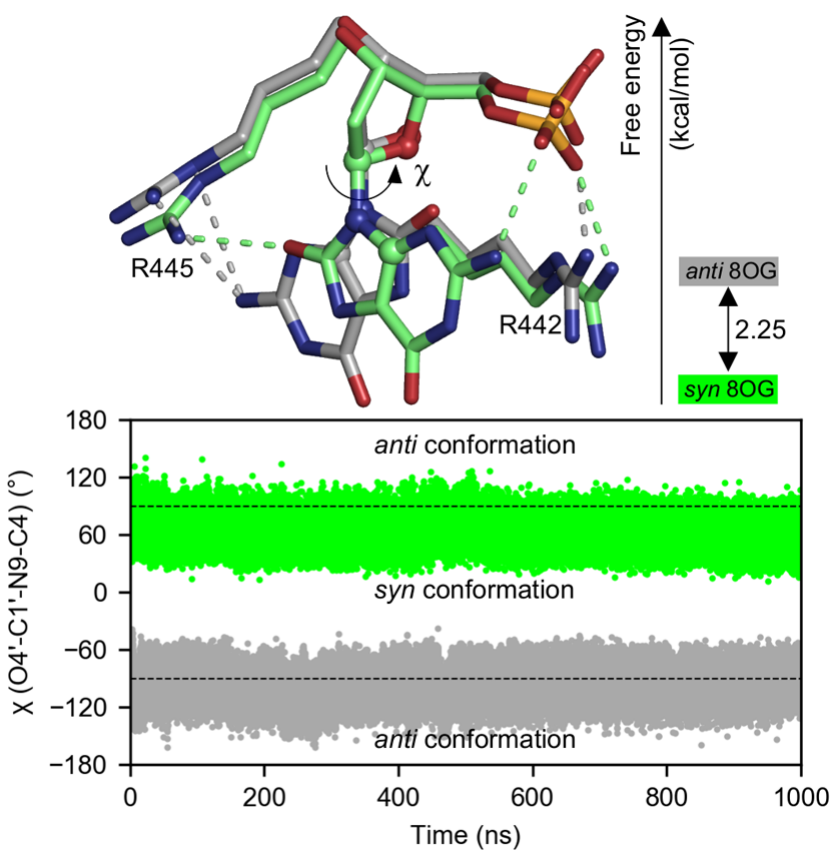
*Anti* and *syn* conformations of 8-oxoguanine (8OG) in the binary DNA/polymerase μ complex. The gray and green dashed lines represent the hydrogen-bond interactions of *anti* 8OG and *syn* 8OG, respectively, with R442 and R445. The time evolution of the glycosyl torsion angle [χ (O4′-C1′–N9-C4), shown in ball-and-stick representation] during the 1-μs unbiased MD simulations is shown. The free energy difference between the two 8OG conformations calculated by the restrain–free-energy perturbation–release method is 2.25 ± 0.05 kcal/mol.

Two models of the binary 8OG-damaged DNA/Pol μ complex, one with 8OG in the *anti* conformation and the other in the *syn* conformation, were built from the crystal structure (PDB ID 6P1M) and simulated for 1 μs each. The *anti* 8OG and *syn* 8OG models are stable throughout the simulation, with protein backbone RMSDs of 1.8 and 1.8 Å, respectively, and DNA backbone RMSDs of 2.6 and 2.3 Å, respectively (see Figure S10 in the Supporting Information). Moreover, there is no significant structural difference between the two models, with the protein and DNA backbone RMSDs being only 0.9 and 1.0 Å, respectively. During the MD simulations, both *anti* and *syn* 8OG predominantly (∼90% occupancy) adopt α [(*n*-1)O3′–P-O5′-C5′] and γ (O5′-C5′-C4′-C3′) torsion angle conformations of +*synclinical* (+30° to +90°). As a result of this conformation, a phosphate O atom clashes with O8 in *anti* 8OG but forms an intramolecular hydrogen bond with an N2 hydrogen in *syn* 8OG (Figure 4). *Anti* and *syn* 8OG also have similar protein interactions: the phosphate group is hydrogen bonded to R442, while the base moiety (N2 and O8 atoms of *anti* and *syn* 8OG, respectively) is hydrogen bonded to R445. On the other hand, the hydrogen bond between the O8 atom of *syn* 8OG and Q441 observed in the crystal structure is broken during the MD simulations. Importantly, during the 1-μs MD simulations, *anti* and *syn* 8OG retain their respective glycosyl torsion angle conformations, with χ values of −96° ± 13° and 62° ± 12°, respectively (Figure 4). In other words, *anti* 8OG does not spontaneously switch to *syn* 8OG, despite the steric repulsion between O8 and the phosphate group.

A hybrid model with 8OG simultaneously in the *anti* and *syn* conformations was constructed from the equilibrated structures from these unbiased MD simulations (Figure 1B). As in the AT-rich DNA model system, 6, 15, and 6 λ-states were used for the restrain, FEP, and release stages, respectively, which led to good phase-space overlap (Figure S11 in the Supporting Information). *Anti* 8OG and *syn* 8OG maintain their respective conformations and remain within the helix during the transformation (Figures S12–S14 in the Supporting Information), because of restraints on the glycosyl and base-flipping torsion angles (see Scheme 2).

**Figure 5:**
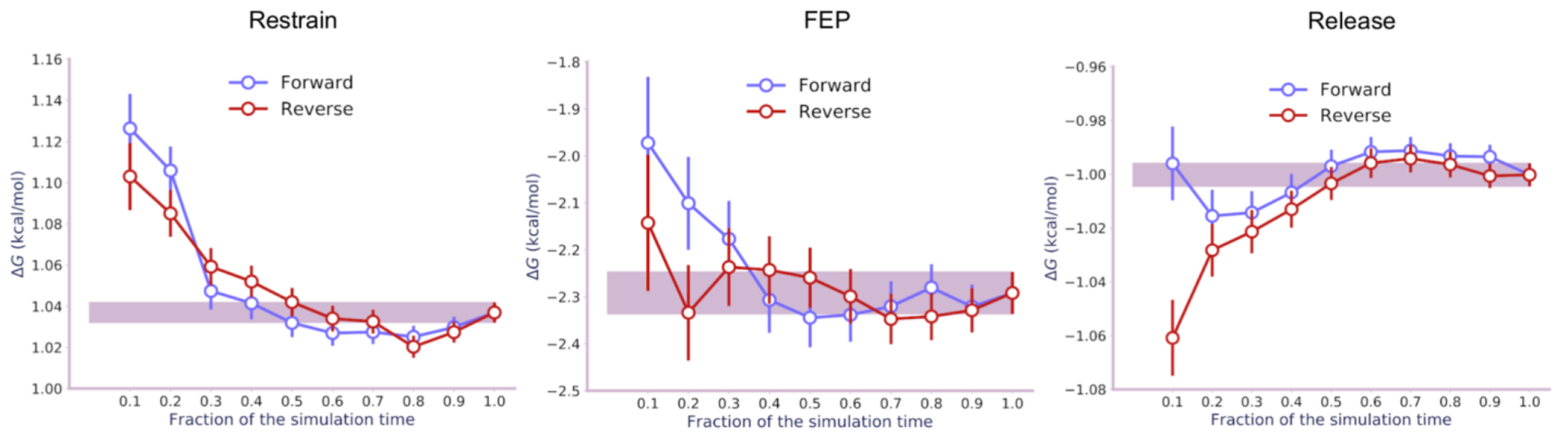
Time evolution of the free energies (with error bars) for the three stages of transformation of 8-oxoguanine from *anti* to *syn* conformation. Data from the last 10 ns of simulations were analyzed both chronologically (“forward”) and in a time-reversed manner (“reverse”).

Figure 5 shows that the restrain, FEP, and release simulations converge in 17, 17, and 16 ns, respectively, leading to a total simulation time of ∼0.5 μs. The R-FEP-R calculations show that *syn* 8OG is lower in energy than *anti* 8OG by 2.25 ± 0.05 kcal/mol (Figure 4). Because *anti* and *syn* 8OG have similar backbone conformations and protein interactions, this free-energy difference can be solely attributed to the change in the glycosyl torsion angle conformation. This result is consistent with the fact that 8OG adopts the *syn* conformation exclusively in the Pol μ binary complex crystal structure (PDB ID 6P1M).^29^ The higher energy of *anti* 8OG can be explained by the steric repulsion between the O8 atom and phosphate group in this conformation. Using the equation Δ*G* = −*RT* ln (*P*_s_/*P*_a_), where *P*_s_ and *P*_a_ are the populations of *syn* and *anti* 8OG, respectively, the relative population *P*_s_/*P*_a_ at 310 K can be estimated as ∼40. The higher population of *syn* 8OG in Pol μ can partially explain the high frequency with which this Pol misincorporates adenine opposite template 8OG. Thus, the R-FEP-R method coupled with the parmbsc1 force field can also accurately predict the conformational preference of purine bases in enzyme-bound DNA, which would be valuable in understanding the factors underlying DNA recognition, replication, and repair by nucleic-acid-processing enzymes.

## Conclusions

In this study, the R-FEP-R/parmbsc1 method was used to predict the base-pairing and conformational preferences of purine bases. In isolated AT-rich DNA, WC base pairing was calculated to be more stable than HG base pairing by 3.13 kcal/mol, which is consistent with NMR relaxation dispersion spectroscopy data. In Pol μ-bound DNA, the *syn* conformation of unpaired 8OG, which leads to HG base pairing with adenine, was calculated to be more stable than the *anti* conformation, by 2.25 kcal/mol, consistent with crystallographic data. The R-FEP-R/parmbsc1 method had a comparable computational cost to metadynamics-based methods but did not require knowledge of the transition pathway between the two end states. With its good accuracy and relatively low computational cost, R-FEP-R/parmbsc1 can be used in conjunction with experimental techniques to verify the occurrence of HG base pairing in free, bound, mismatched, and damaged DNA. Such method would aid in investigating the role of the base pairing mode in the recognition, binding, and damage repair of DNA.

## Supporting Information Available

Computational details and additional data from unbiased MD simulations and R-FEP-R calculations (PDF)
