# Supplementary material for: Alchemical Free-Energy Calculations of Watson–Crick and Hoogsteen Base Pairing Interconversion in DNA
Source: J Chem Theory Comput. 2022 Oct 6;18(11):6966–73. doi: 10.1021/acs.jctc.2c00848 (PMC9648191; doi:10.1021/acs.jctc.2c00848)
Supplement: Supplementary file 1 — ct2c00848_si_001.pdf [file ct2c00848_si_001.pdf]

# SUPPORTING INFORMATION

# Alchemical Free Energy Calculations of Watson–Crick and Hoogsteen Base Pairing Interconversion in DNA

Inacrist Geronimo and Marco De Vivo*

Laboratory of Molecular Modelling & Drug Discovery, Istituto Italiano di Tecnologia, Via Morego 30, Genoa 16163, Italy

***Corresponding author:**

Marco De Vivo, Email: marco.devivo@iit.it

**General unbiased MD simulation protocol.** The systems were minimized for 1000 steps using the steepest descent algorithm, followed by the conjugate gradient algorithm, until the maximum force was less than 100 kJ $mol^{-1}$ $nm^{-1}$. Subsequently, the systems were heated to 300 K (isolated AT-rich DNA) or 310 K (binary 8-oxoguanine (8OG)-damaged DNA/polymerase μ complex) for 100 ps in the NVT ensemble and equilibrated for 500 ps in the NPT ensemble with all heavy atoms restrained. Constant temperature was maintained using Langevin dynamics[1] with a time coupling constant of 2 ps. A constant pressure of 1 bar was maintained using the Berendsen algorithm[2] during equilibration, and the Parrinello–Rahman algorithm[3] during production with a time coupling constant of 2 ps. Periodic boundary conditions were applied, and long-range electrostatic interactions were calculated using the particle mesh Ewald method[4] with a real-space cut-off of 12 Å. Bonds with hydrogen were constrained using the LINCS algorithm,[5] which allowed a time step of 2 fs.

**Charge derivation for 8OG.** Geometry optimization of the *anti* and *syn* conformations of 8OG at the MP2/6-31G* level and electrostatic potential calculations at the HF/6-31G* level were performed using Gaussian 09.[6] Subsequently, a multi-conformational restrained electrostatic potential[7,8] fitting over the two 8OG conformations was performed using antechamber.[9] Only the charges of the purine, C1′, and H1′ atoms were derived, while the rest of the atoms were constrained to have the same charges as those in undamaged deoxyguanosine (Table S1).

**Table S1.** Derived partial charges of 8-oxoguanine.[a]

| Atom name | Atom type | Charge | Atom name | Atom type | Charge |
|---|---|---|---|---|---|
| N9 | N* | -0.004566 | H1 | H | 0.348606 |
| C8 | C | 0.408236 | C2 | CA | 0.717325 |
| O8 | O | -0.490468 | N2 | N2 | -0.811768 |
| N7 | NA | -0.548021 | H21 | H | 0.372421 |
| H7 | H | 0.398226 | H22 | H | 0.372421 |
| C5 | CB | 0.007740 | N3 | NC | -0.529776 |
| C6 | C | 0.609879 | C4 | CB | 0.106197 |
| O6 | O | -0.625336 | C1′ | CT | 0.543252 |
| N1 | NA | -0.576626 | H1′ | H2 | -0.176142 |

[a] For the other atoms, the atom types and charges are the same as those of deoxyguanosine.

```
; residue    5 8AS rtp 8AS q -1.2
  125    P      5   8AS   P     125   1.165900    30.9700
  126   O2      5   8AS   OP1   126  -0.776100    16.0000
  127   O2      5   8AS   OP2   127  -0.776100    16.0000
  128   OS      5   8AS   O5'   128  -0.495400    16.0000
  129   CI      5   8AS   C5'   129  -0.006900    12.0100
  130   H1      5   8AS   H5'   130   0.075400     1.0080
  131   H1      5   8AS   H5''  131   0.075400     1.0080
  132   CT      5   8AS   C4'   132   0.162900    12.0100
  133   H1      5   8AS   H4'   133   0.117600     1.0080
  134   OS      5   8AS   O4'   134  -0.369100    16.0000   DUM_OS    0.000000    1.0000
  135   CT      5   8AS   C1'   135   0.543252    12.0100   DUM_CT    0.000000    1.0000
  136   H2      5   8AS   H1'   136  -0.176142     1.0080   DUM_H2    0.000000    1.0000
  137   N*      5   8AS   N9    137  -0.004566    14.0100   DUM_N*    0.000000    1.0000
  138   C       5   8AS   C8    138   0.408236    12.0100   DUM_C     0.000000    1.0000
  139   O       5   8AS   O8    139  -0.490468    16.0000   DUM_O     0.000000    1.0000
  140   NA      5   8AS   N7    140  -0.548021    14.0100   DUM_NA    0.000000    1.0000
  141   H       5   8AS   H7    141   0.398226     1.0080   DUM_H     0.000000    1.0000
  142   CB      5   8AS   C5    142   0.007740    12.0100   DUM_CB    0.000000    1.0000
  143   C       5   8AS   C6    143   0.609879    12.0100   DUM_C     0.000000    1.0000
  144   O       5   8AS   O6    144  -0.625336    16.0000   DUM_O     0.000000    1.0000
  145   NA      5   8AS   N1    145  -0.576626    14.0100   DUM_NA    0.000000    1.0000
  146   H       5   8AS   H1    146   0.348606     1.0080   DUM_H     0.000000    1.0000
  147   CA      5   8AS   C2    147   0.717325    12.0100   DUM_CA    0.000000    1.0000
  148   N2      5   8AS   N2    148  -0.811768    14.0100   DUM_N2    0.000000    1.0000
  149   H       5   8AS   H21   149   0.372421     1.0080   DUM_H     0.000000    1.0000
  150   H       5   8AS   H22   150   0.372421     1.0080   DUM_H     0.000000    1.0000
  151   NC      5   8AS   N3    151  -0.529776    14.0100   DUM_NC    0.000000    1.0000
  152   CB      5   8AS   C4    152   0.106197    12.0100   DUM_CB    0.000000    1.0000
  153   CE      5   8AS   C3'   153   0.071300    12.0010
  154   H1      5   8AS   H3'   154   0.098500     1.0080
  155   CT      5   8AS   C2'   155  -0.085400    12.0100   DUM_CT    0.000000    1.0000
  156   HC      5   8AS   H2'   156   0.071800     1.0080   DUM_HC    0.000000    1.0000
  157   HC      5   8AS   H2''  157   0.071800     1.0080   DUM_HC    0.000000    1.0000
  158   OS      5   8AS   O3'   158  -0.523200    16.0000
  159   DUM_OS  5   8AS   DO4'  159   0.000000     1.0000   OS       -0.369100   16.0000
  160   DUM_CT  5   8AS   DC1'  160   0.000000     1.0000   CT        0.543252   12.0100
  161   DUM_H2  5   8AS   DH1'  161   0.000000     1.0000   H2       -0.176142    1.0080
  162   DUM_N*  5   8AS   DN9   162   0.000000     1.0000   N*       -0.004566   14.0100
  163   DUM_C   5   8AS   DC8   163   0.000000     1.0000   C         0.408236   12.0100
  164   DUM_O   5   8AS   DO8   164   0.000000     1.0000   O        -0.490468   16.0000
  165   DUM_NA  5   8AS   DN7   165   0.000000     1.0000   NA       -0.548021   14.0100
  166   DUM_H   5   8AS   DH7   166   0.000000     1.0000   H         0.398226    1.0080
  167   DUM_CB  5   8AS   DC5   167   0.000000     1.0000   CB        0.007740   12.0100
  168   DUM_C   5   8AS   DC6   168   0.000000     1.0000   C         0.609879   12.0100
  169   DUM_O   5   8AS   DO6   169   0.000000     1.0000   O        -0.625336   16.0000
  170   DUM_NA  5   8AS   DN1   170   0.000000     1.0000   NA       -0.576626   14.0100
  171   DUM_H   5   8AS   DH1   171   0.000000     1.0000   H         0.348606    1.0080
  172   DUM_CA  5   8AS   DC2   172   0.000000     1.0000   CA        0.717325   12.0100
  173   DUM_N2  5   8AS   DN2   173   0.000000     1.0000   N2       -0.811768   14.0100
  174   DUM_H   5   8AS   DH21  174   0.000000     1.0000   H         0.372421    1.0080
  175   DUM_H   5   8AS   DH22  175   0.000000     1.0000   H         0.372421    1.0080
  176   DUM_NC  5   8AS   DN3   176   0.000000     1.0000   NC       -0.529776   14.0100
  177   DUM_CB  5   8AS   DC4   177   0.000000     1.0000   CB        0.106197   12.0100
  178   DUM_CT  5   8AS   DC2'  178   0.000000     1.0000   CT       -0.085400   12.0100
  179   DUM_HC  5   8AS   DH23  179   0.000000     1.0000   HC        0.071800    1.0080
  180   DUM_HC  5   8AS   DH24  180   0.000000     1.0000   HC        0.071800    1.0080
```

**Figure S1.** `[ atoms ]` Directive of the GROMACS topology file showing the transformation of real atoms to virtual atoms and vice versa. When the atom is virtual, the atom type is prefixed by "DUM_" and the charge is zero.

**Table S2.** Reference glycosyl torsion angles, H-bond distances, and H-bond angles for the harmonic restraints obtained from unbiased MD simulations of isolated AT-rich DNA with Watson–Crick (WC) or Hoogsteen (HG) base pairing modes of A4:T9 (see Scheme 2).

| | WC | HG |
|---|---|---|
| Glycosyl torsion angle | -102.6 | 64.9 |
| H-bond distance (Å)/angle (°) | | |
| $hb_{WC}$ | 3.0/10.9 | - |
| $hb_{HG}$ | - | 3.1/10.9 |
| $hb_{C}$ | 3.0/11.4 | 2.9/14.3 |

**Table S3.** Reference torsion angles for the harmonic restraints obtained from unbiased MD simulations of the DNA polymerase μ binary complex with *anti* or *syn* 8-oxoguanine (see Scheme 2).

| Torsion angle | *anti* | *syn* |
|---|---|---|
| Glycosyl (°) | -97.8 | 64.8 |
| Base-flipping (°) | 2.8 | 9.1 |

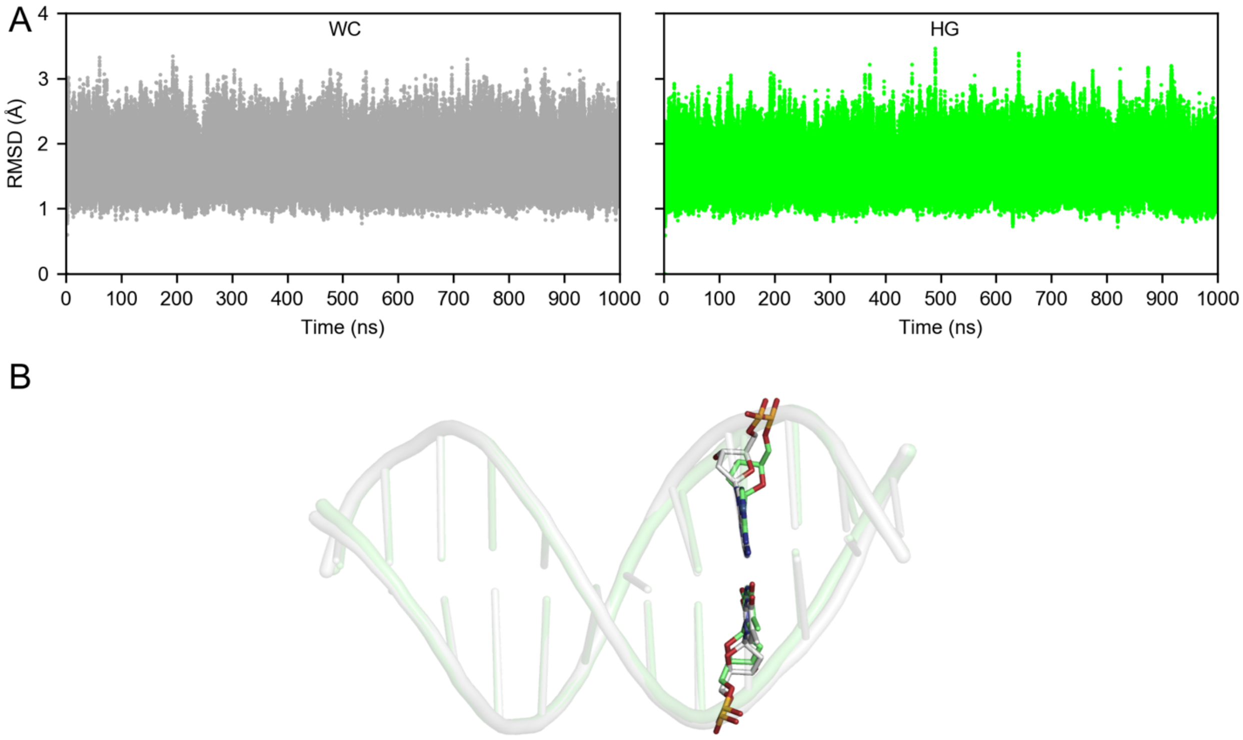


**Figure S2.** (A) Time evolution of backbone RMSDs in isolated AT-rich DNA with Watson–Crick (WC, grey) and Hoogsteen (HG, green) base pairing modes of A4:T9. Terminal base pairs were excluded from the calculation. (B) Comparison of the two structures from unbiased MD simulations.

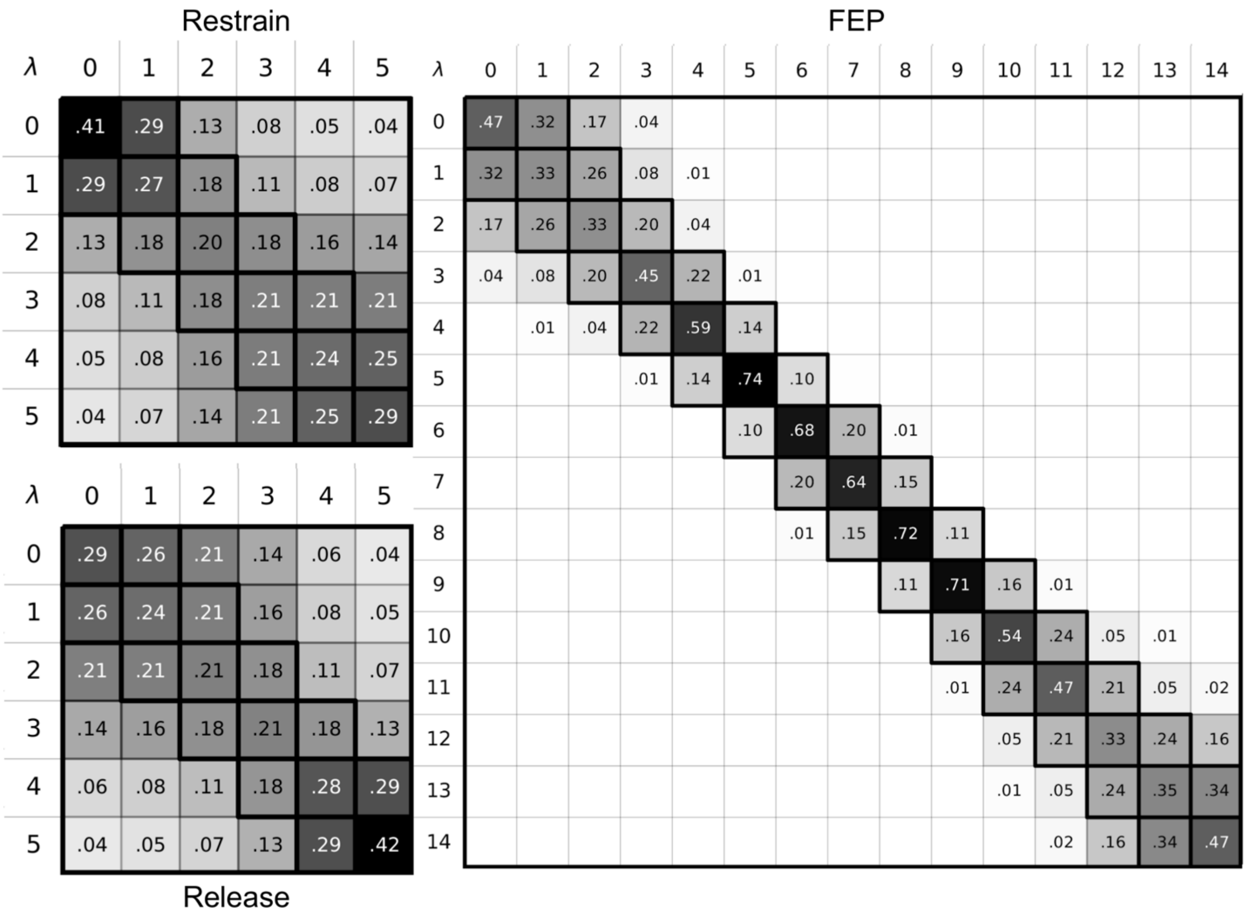


**Figure S3.** Overlap matrices for the three stages of transformation of A4:T9 from Watson–Crick to Hoogsteen base pairing. The element O$ij$ is the probability of observing a sample from state $i$ ($i^{th}$ row) in state $j$ ($j^{th}$ column). The recommended minimum probability for adjacent states (highlighted by thick black lines) is 0.03.[10]

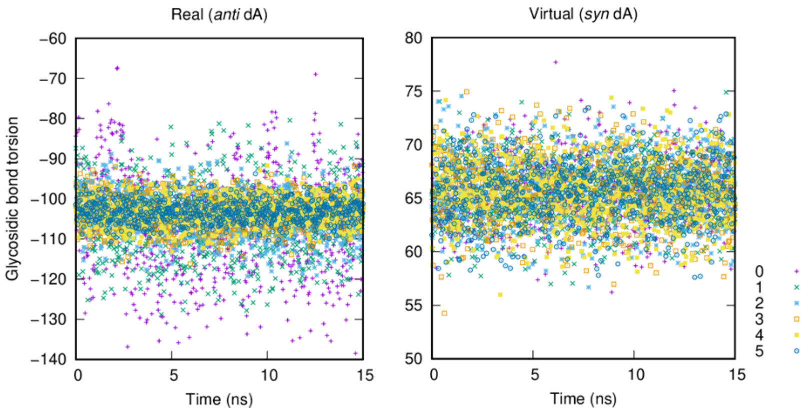


**Figure S4.** Time evolution of the glycosyl torsion angles of *anti* and *syn* A4 at all $\lambda$-states during the "restrain" stage of the transformation (data collected every 20 ps). The harmonic restraints on the real atoms were switched on from state 0 to state 5, while those on the virtual atoms were on at all $\lambda$-states.

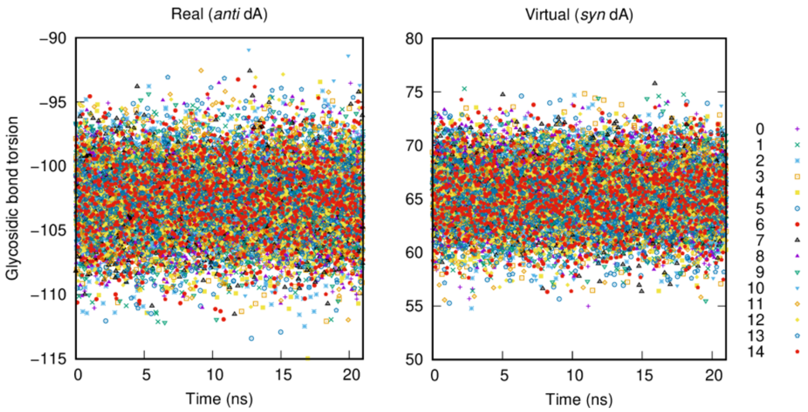


**Figure S5.** Time evolution of the glycosyl torsion angles of *anti* and *syn* A4 at all $\lambda$-states during the "FEP" stage of the transformation (data collected every 20 ps). The harmonic restraints on both real and virtual atoms were on at all $\lambda$-states.

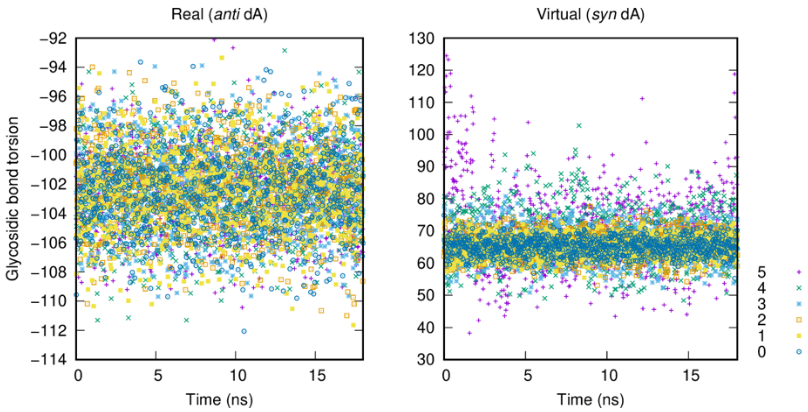


**Figure S6.** Time evolution of the glycosyl angles of *anti* and *syn* A4 at all $\lambda$-states during the "release" stage of the transformation (data collected every 20 ps). The harmonic restraints on the virtual atoms were switched off from state 0 to state 5, while those on the real atoms were on at all $\lambda$-states.

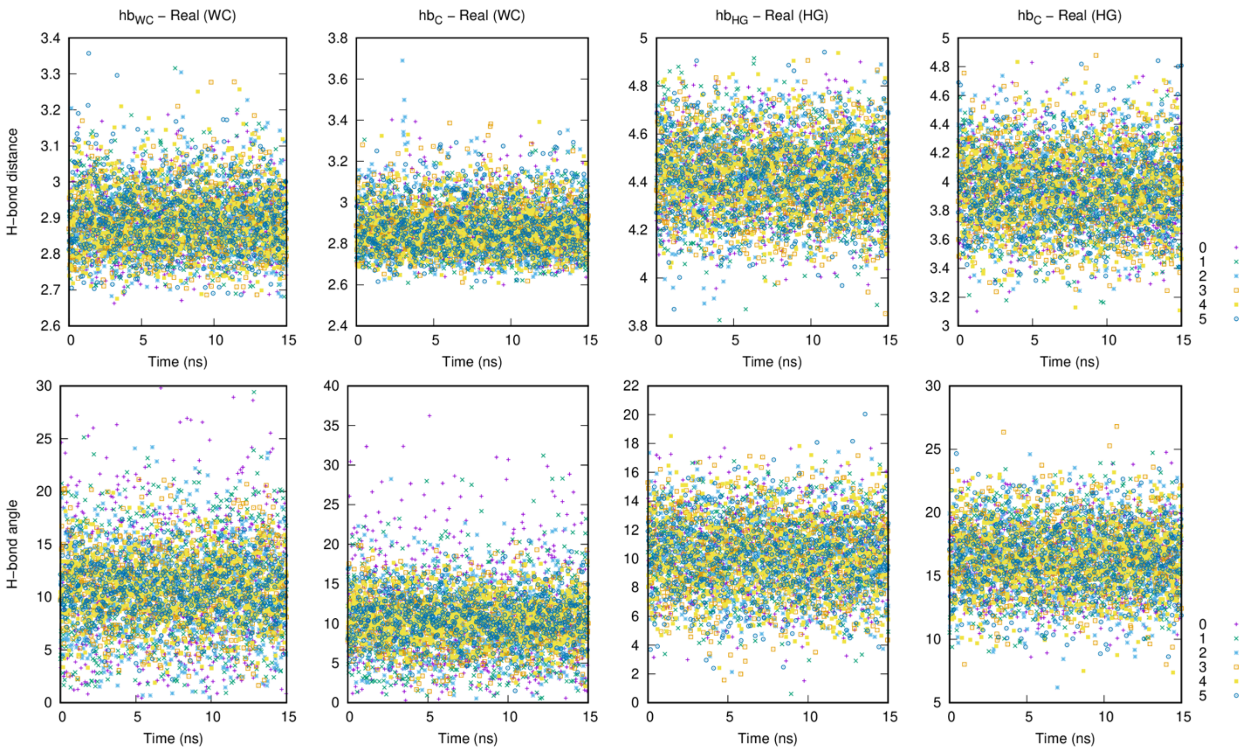


**Figure S7.** Time evolution of the H-bond distances and angles in the Watson–Crick (WC) and Hoogsteen (HG) base pairing modes of A4:T9 (see Scheme 2) at all $\lambda$-states during the “restrain” stage of the transformation (data collected every 20 ps). The harmonic restraints on the real atoms were switched on from state 0 to state 5, while those on the virtual atoms were on at all $\lambda$-states.

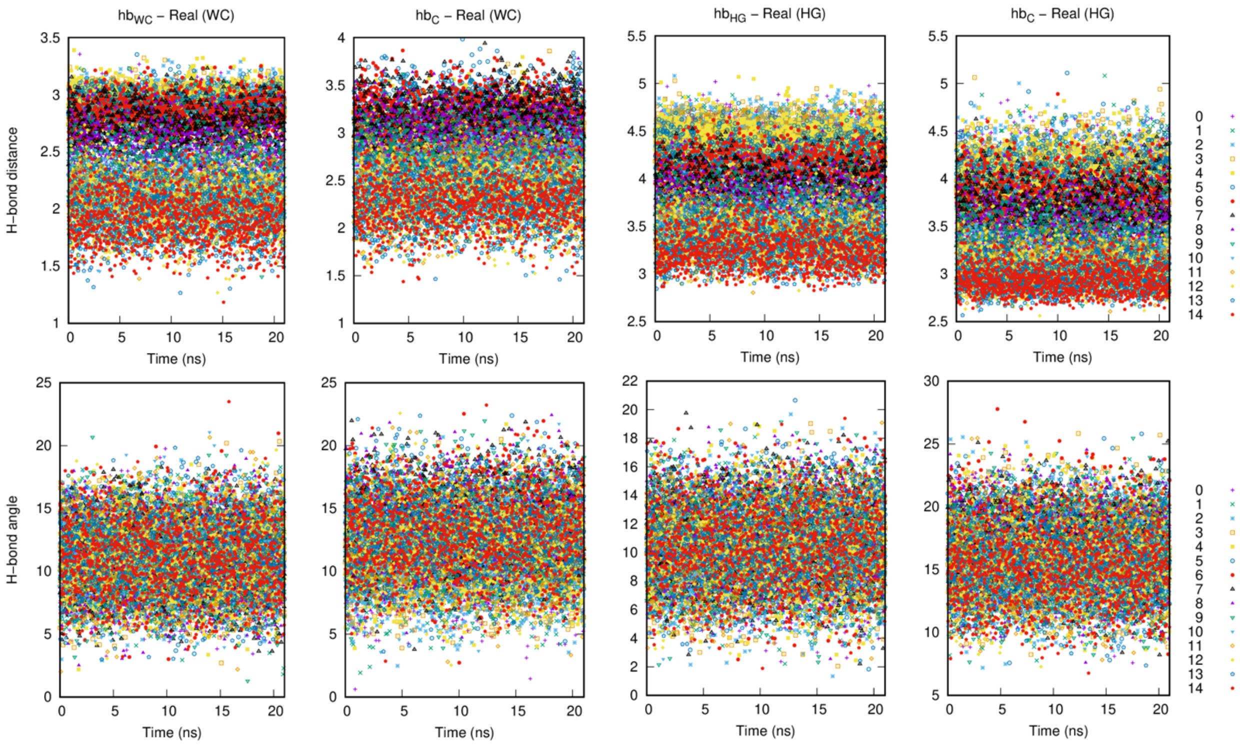


**Figure S8.** Time evolution of the H-bond distances and angles in the Watson–Crick (WC) and Hoogsteen (HG) base pairing modes of A4:T9 (see Scheme 2) at all $\lambda$-states during the “FEP” stage of the transformation (data collected every 20 ps). The harmonic restraints on both real and virtual atoms were on at all $\lambda$-states.

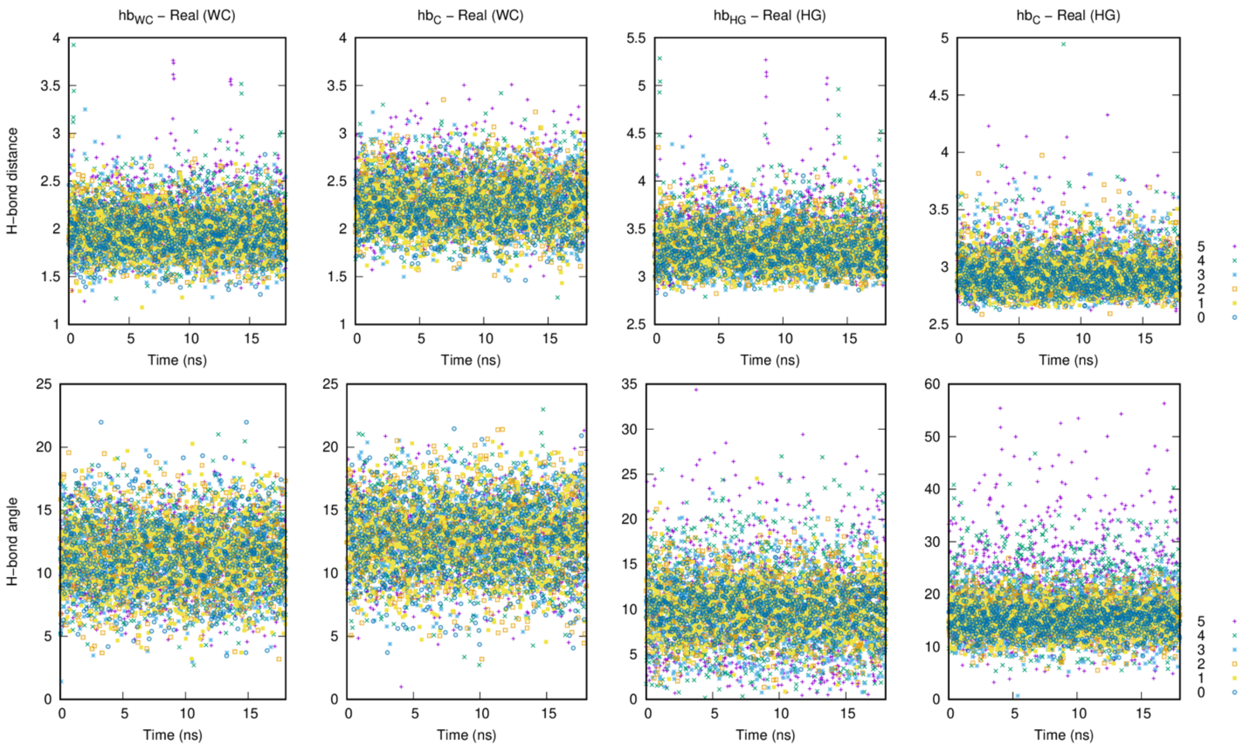


**Figure S9.** Time evolution of the H-bond distances and angles in the Watson–Crick (WC) and Hoogsteen (HG) base pairing modes of A4:T9 (see Scheme 2) at all $\lambda$-states during the "release" stage of the transformation (data collected every 20 ps). The harmonic restraints on the virtual atoms were switched off from state 0 to state 5, while those on the real atoms were on at all $\lambda$-states.

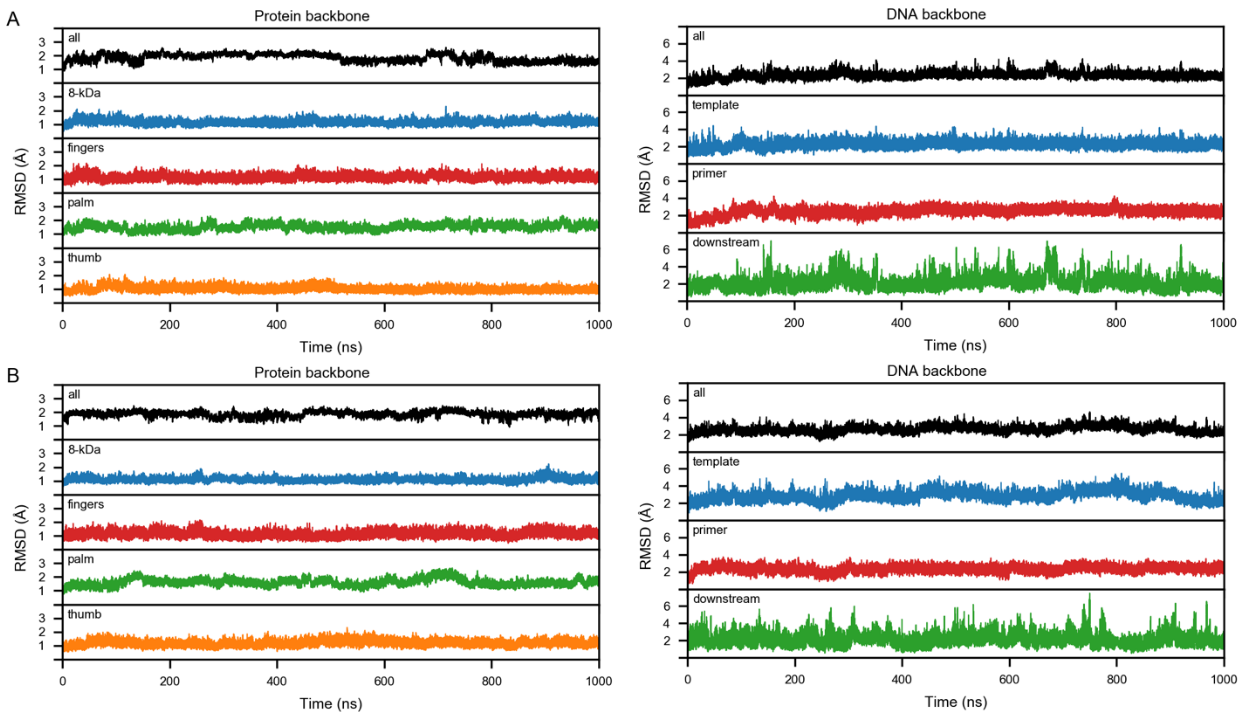


**Figure S10.** Time evolution of backbone RMSDs in the DNA polymerase μ binary complex with 8-oxoguanine in the (A) *syn* and (B) *anti* conformations. The flexible loop 1 (C369–F385), which was missing from the crystal structure and added by modeling, was excluded from the calculation.

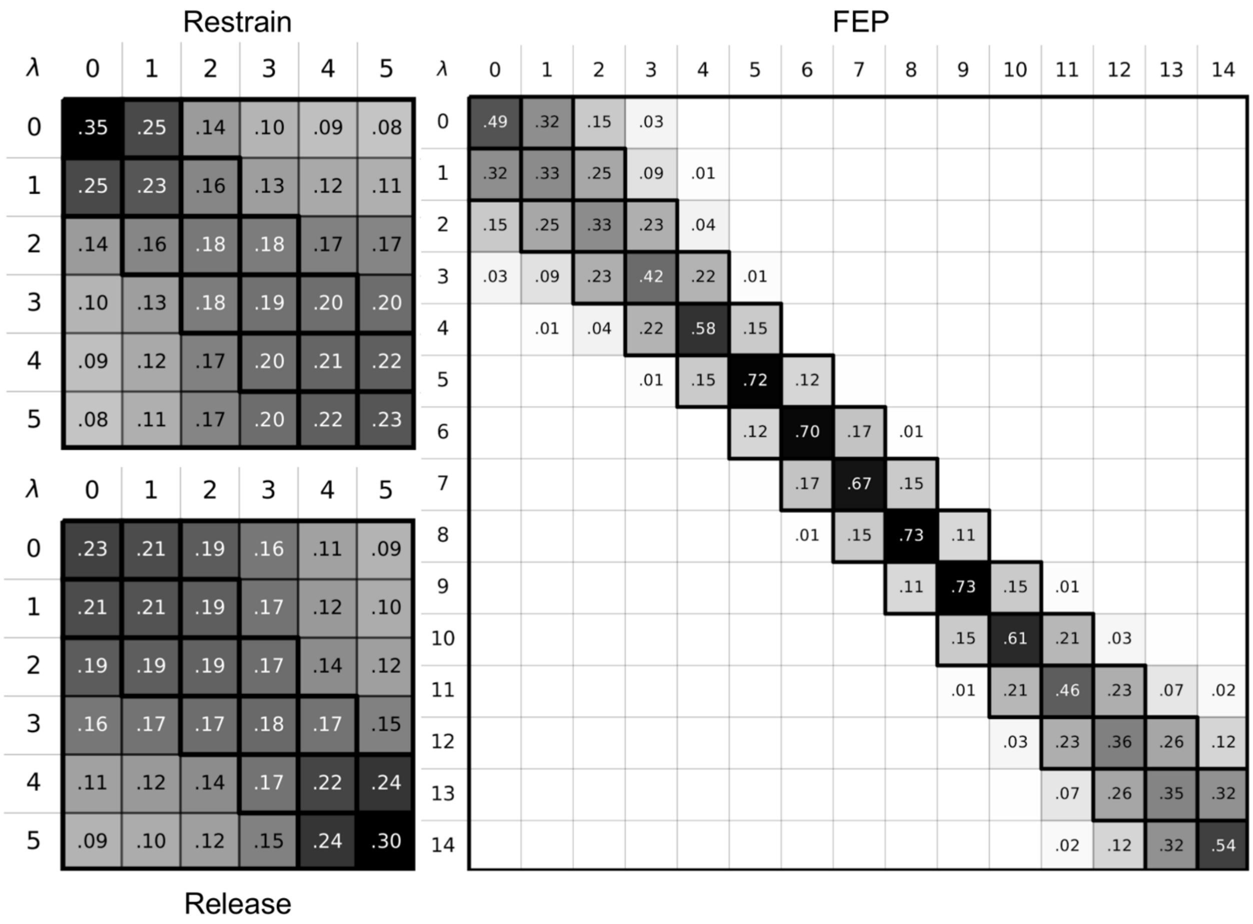


**Figure S11.** Overlap matrices for the three stages of transformation of 8-oxoguanine from *anti* to *syn* conformation. The element O*ij* is the probability of observing a sample from state *i* ($i^{th}$ row) in state *j* ($j^{th}$ column). The recommended minimum probability for adjacent states (highlighted by thick black lines) is 0.03.[10]

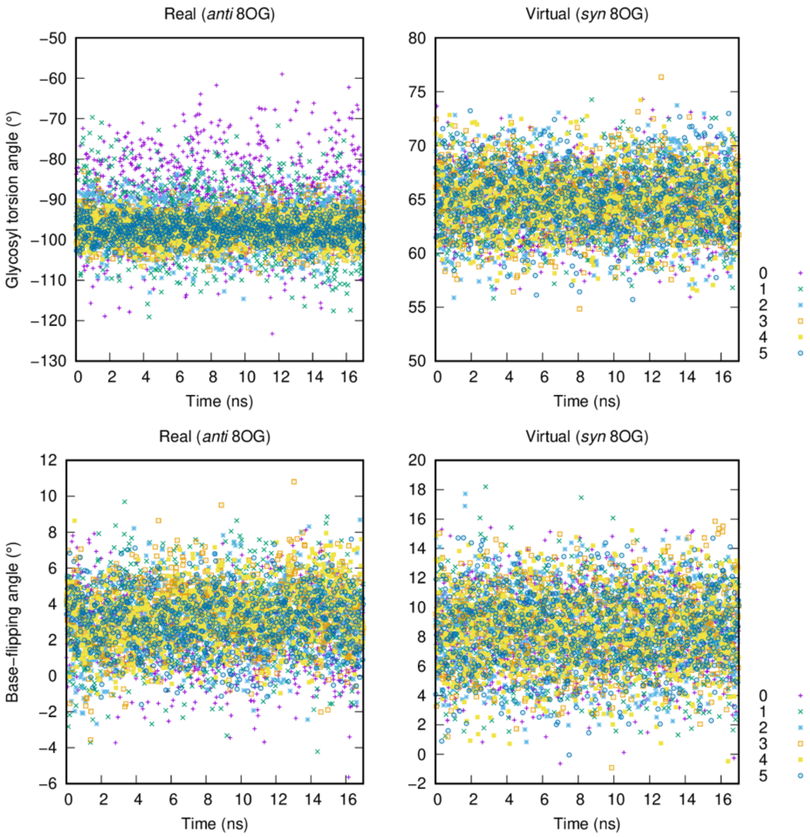


**Figure S12.** Time evolution of the glycosyl and base-flipping torsion angles of *anti* and *syn* 8-oxoguanine (8OG) at all $\lambda$-states during the "restrain" stage of the transformation (data collected every 20 ps). The harmonic restraints on the real atoms were switched on from state 0 to state 5, while those on the virtual atoms were on at all $\lambda$-states.

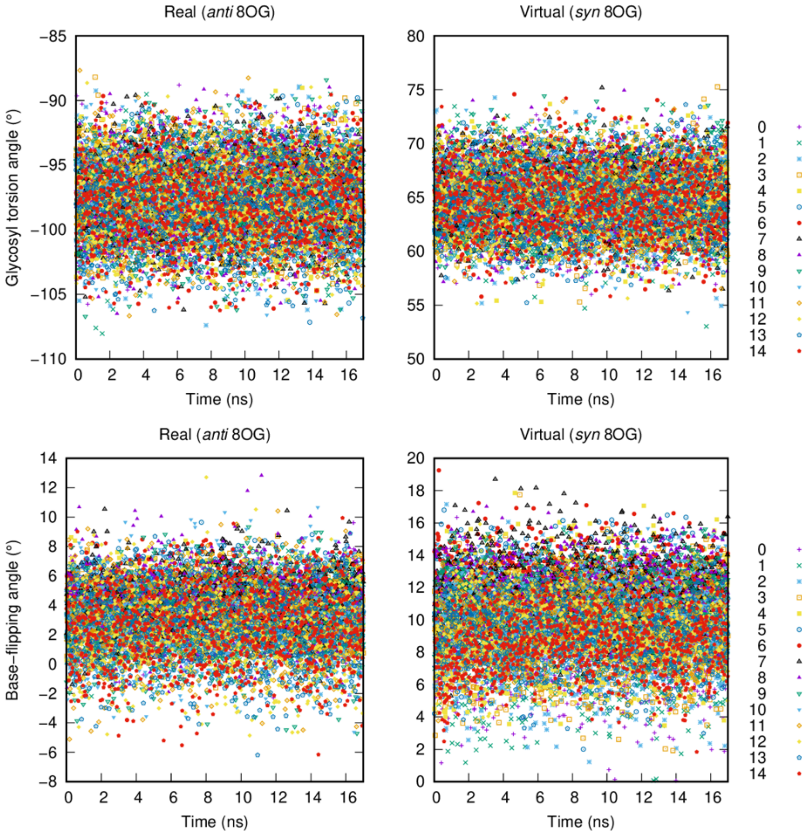


**Figure S13.** Time evolution of the glycosyl and base-flipping torsion angles of *anti* and *syn* 8-oxoguanine (8OG) at all $\lambda$-states during the "FEP" stage of the transformation (data collected every 20 ps). The harmonic restraints on both real and virtual atoms were on at all $\lambda$-states.

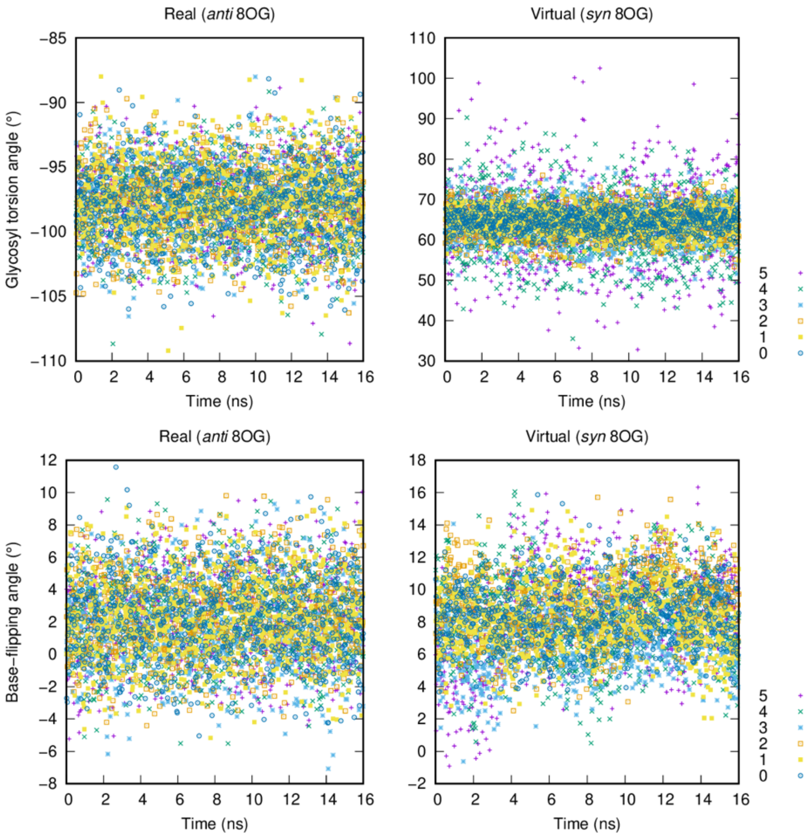


**Figure S14.** Time evolution of the glycosyl and base-flipping torsion angles of *anti* and *syn* 8-oxoguanine (8OG) at all $\lambda$-states during the "release" stage of the transformation (data collected every 20 ps). The harmonic restraints on the virtual atoms were switched off from state 0 to state 5, while those on the real atoms were on at all $\lambda$-states.

## References


(1) Pastor, R. W.; Brooks, B. R.; Szabo, A. An Analysis of the Accuracy of Langevin and Molecular Dynamics Algorithms. *Mol. Phys.* **1988**, *65*, 1409–1419.

(2) Berendsen, H. J. C.; Postma, J. P. M.; van Gunsteren, W. F.; DiNola, A.; Haak, J. R. Molecular Dynamics with Coupling to an External Bath. *J. Chem. Phys.* **1984**, *81*, 3684–3690.

(3) Parrinello, M.; Rahman, A. Polymorphic Transitions in Single Crystals: A New Molecular Dynamics Method. *J. Appl. Phys.* **1981**, *52*, 7182–7190.

(4) Essmann, U.; Perera, L.; Berkowitz, M. L.; Darden, T.; Lee, H.; Pedersen, L. G. A Smooth Particle Mesh Ewald Method. *J. Chem. Phys.* **1995**, *103*, 8577–8593.

(5) Hess, B.; Bekker, H.; Berendsen, H. J. C.; Fraaije, J. G. E. M. LINCS: A Linear Constraint Solver for Molecular Simulations. *J. Comput. Chem.* **1997**, *18*, 1463–1472.

(6) Frisch, M. J.; Trucks, G. W.; Schlegel, H. B.; Scuseria, G. E.; Robb, M. A.; Cheeseman, J. R.; Scalmani, G.; Barone, V.; Petersson, G. A.; Nakatsuji, H.; et al. Gaussian 09, Revision A.02. Gaussian, Inc.: Wallingford CT 2016.

(7) Bayly, C. I.; Cieplak, P.; Cornell, W.; Kollman, P. A. A Well-Behaved Electrostatic Potential Based Method Using Charge Restraints for Deriving Atomic Charges: The RESP Model. *J. Phys. Chem.* **1993**, *97*, 10269–10280.

(8) Cieplak, P.; Cornell, W. D.; Bayly, C.; Kollman, P. A. Application of the Multimolecule and

Multiconformational RESP Methodology to Biopolymers: Charge Derivation for DNA, RNA, and Proteins. *J. Comput. Chem.* **1995**, *16*, 1357–1377.
(9) Wang, J.; Wang, W.; Kollman, P. A.; Case, D. A. Automatic Atom Type and Bond Type Perception in Molecular Mechanical Calculations. *J. Mol. Graph. Model.* **2006**, *25*, 247–260.
(10) Klimovich, P. V; Shirts, M. R.; Mobley, D. L. Guidelines for the Analysis of Free Energy Calculations. *J. Comput. Aided. Mol. Des.* **2015**, *29*, 397–411.
